# Standard versus innovative robotic balance assessment for people with multiple sclerosis: a correlational study

**DOI:** 10.1186/s40001-023-01223-2

**Published:** 2023-07-26

**Authors:** Jessica Podda, Giorgia Marchesi, Valentina Squeri, Alice De Luca, Alice Bellosta, Ludovico Pedullà, Giovanna Konrad, Mario Alberto Battaglia, Giampaolo Brichetto, Andrea Tacchino

**Affiliations:** 1 Scientific Research Area, Italian Multiple Sclerosis Foundation (FISM), Via Operai 40, 16149 Genoa, Italy; 2 Movendo Technology S.R.L, Genoa, Italy; 3 AISM Rehabilitation Service, Italian Multiple Sclerosis Society, Genoa, Italy; 4 Department of Physiopathology, Experimental Medicine and Public Health, University of Siena, Siena, Italy

**Keywords:** Multiple sclerosis, Balance, Hunova®, EquiTest®, Posturography

## Abstract

**Introduction:**

Balance disorders are common in people with Multiple Sclerosis (PwMS) and, together with other impairments and disabilities, often prevent PwMS from performing their daily living activities. Besides clinical scales and performance tests, robotic platforms can provide more sensitive, specific, and objective monitoring. Validated technologies have been adopted as gold standard, but innovative robotic solutions would represent an opportunity to detect balance impairment in PwMS.

**Aim:**

Study’s aim was to compare postural assessment of 46 PwMS with a relapsing–remitting form during static tasks performed with the novel robotic platform hunova® and the gold standard EquiTest®,

**Methods:**

Pearson’s r was run on Center of Pressure (COP)-related parameters and global static balance measures computed from hunova® and EquiTest® in eyes-open (EO) and eyes-closed (EC) conditions. In addition, agreeableness level toward the use of both devices was tested through numeric rating scale.

**Results:**

Considering COP-related parameters, correlations were significant for all measures (*p* < .001). Interestingly, in EO, a strong correlation was shown for sway area (*r* = .770), while Medio-Lateral (ML) and Anterior–Posterior (AP) oscillation range, path length, ML and AP speed, ML and AP root mean square distance had a relatively strong association (.454 ≤ *r* ≤ .576). In EC, except for ML oscillation range showing a relatively strong correlation (r = .532), other parameters were strongly associated (.603 ≤ *r* ≤ .782). Correlations between global balance indexes of hunova® and EquiTest® revealed a relatively strong association between the Somatosensory Score in EquiTest® and the Somatosensory Index in hunova® (*r* = − .488). While in EO Static Balance Index from hunova® was highly correlated with Equilibrium score of EquiTest® (*r* = .416), Static Balance Index had a relatively strong association with both the Equilibrium (*r* = .482) and Strategy Score (*r* = .583) of EquiTest® in EC. Results from agreeableness rating scale revealed that hunova® was highly appreciated compared to EquiTest® (*p* = .044).

**Conclusions:**

hunova® represents an innovative adjunct to standard robotic balance evaluation for PwMS. This confirms that combining traditional and robotic assessments can more accurately detect balance impairments in MS.

**Supplementary Information:**

The online version contains supplementary material available at 10.1186/s40001-023-01223-2.

## Background

Multiple sclerosis (MS) is a chronic inflammatory and degenerative disease of the central nervous system (CNS) that can result in significant physical and mental symptoms, especially abnormal walking, muscle weakness, spasticity, fatigue, cognitive impairments, and mood disorders. In addition, swing during quite standing, moving slowly following postural disturbances and inability to maintain balance are common in MS. These impairments often prevent people with MS (PwMS) from performing their daily living activities and are also risk factors for falls [1, 2].

The control of balance relies on the complex integration of information from the somatosensory, vestibular, and visual systems, which work together with the neuromuscular system to maintain an upright posture over a base of support (static balance) or stability during walking (dynamic balance) [3]. A complete characterization of the balance deficits due to MS is a key factor to monitor disease progression, prevent falls and especially to tailor rehabilitative interventions, as new information can be beneficial to clinicians in terms of assessment and prognosis of disabilities in the MS population. Several validated scales and clinical tests, such as the Timed-Up-and-Go (TUG) Test [4, 5], Berg Balance Scale (BBS) [5, 6], Mini-Balance Evaluation Systems TesT (Mini-BESTest) [7] and The Activities-specific Balance Confidence (ABC) Scale [8], are often used in the MS clinical setting. Clinical tests usually rate balance performance on a set of motor tasks, where scoring is based on the sum of ordinal item scores or stopwatch measurements. However, although these scales are easy and relatively quick to use, they have been shown to suffer from ceiling and/or flooring effects [9] and to hold good specificity but limited sensitivity in PwMS [10]. Furthermore, they are hampered by their variable execution and by the room left for evaluator judgment in the scoring system [11]. Parallel to clinical scales, posturography can provide valuable information about an individual's postural stability, including ability to maintain balance in different sensory conditions (e.g., standing with eyes open or closed, on a stable or unstable surface), postural sway patterns, and response to sensory perturbations (e.g., sudden movements of the platform). In addition, posturography can detect changes in balance that may not be apparent to either the physician or the individual being evaluated. In this context, a large variety of technological instrumental tests are used to evaluate postural stability in both static and dynamic tasks. Force platforms are one such tool that measures ground reaction forces generated by the body to evaluate biomechanical aspects of balance control [11]. The same information can also be obtained by pressure-sensitive systems and electromechanical platforms. All these devices allows the computation of center of pressure (COP)-related balance measures, such as sway parameters that have been shown to change significantly in MS [11–13]. In quiet stance, the COP is estimated as compatible with the center of gravity at about 97%. Variations in the instant positions of the COP during a 30- or 60-s test are used to calculate time-domain measures, including the velocity of the COP on the anteroposterior or mediolateral axes (mm/s), the sum of the displacements (path) of COP (mm), and the 95% confidence ellipse area of COP (mm^2^) [11]. Moreover, COP-related parameters can be used to better discriminate among PwMS with different levels of balance impairments and involved in the evaluation of the effect of neuro-rehabilitative balance interventions [11, 14]. Furthermore, posturography in PwMS has been proved to explore the relationship between balance and disability level, since consistent evidence indicates that as the neurological disability increases, the postural balance is progressively impaired. PwMS with higher disability needed a larger area for standing than healthy subjects or people with a lower score, suggesting that postulated growing standing instability with an increase in the severity of clinical impairment [12, 15, 16]. Kalron and colleagues found that PwMS with an Expanded Disability Status Scale (EDSS) score of 6.0–6.5 were significantly poorer in traditional balance measures as CoP path length, sway area in both open, and closed eyes conditions compared to other disability subgroups [12].

To date, the EquiTest® (NeuroCom International, Inc., Clackamas, OR) is considered a *gold standard* to assess both static and dynamic postural stability and balance in healthy (e.g., children, elderly, and military personnel) [17–19] and neurological (e.g., mild traumatic brain injury, Parkinson disease, Alzheimer disease, and PwMS) [14, 20–22] populations. However, although its assessment value, EquiTest® may be limited due to several factors as its reliance on only sagittal plane movements and on the issue that NeuroCom balance manager systems will be no longer available (its support will be discontinued in 2026), suggesting that EquiTest® will eventually be gone from clinics [13].

In this context, the development of new advanced robotic platforms, usually adopted to deliver balance rehabilitative exercises, would represent a new opportunity to perform a balance assessment by allowing observing a wider array of postural characteristics. Indeed, the use of robotic platforms has been growing rapidly by laying the foundation for the improvement not only of the rehabilitative interventions effectiveness (e.g., massed practice, task specificity and personalization of the difficulty levels) [3, 23], but also of the clinical assessment (e.g., in sensitivity and specificity) [14, 24]. hunova® (Movendo Technology s.r.l., Genoa, Italy) [23] is an advanced robotic system designed and developed with the goal of covering a considerable amount of activities, typical of physical therapy, that enables the evaluation of traditional stabilometric parameters and allows the implementation of different dynamic environments that stimulate postural responses. Due to these considerations, the objective was to investigate whether hunova® could be used to expand the computerized posturography balance analysis and thus may represent a valid alternative to the EquiTest®. Here, as first step toward this aim, we compare in PwMS the postural assessment during static tasks performed with hunova® and the gold standard EquiTest®. By starting from the raw data recorded during tasks consisting in standing with eyes open (EO) or close (EC), we computed and compared COP balance parameters and composite indexes calculated as global measures of balance. More precisely, first, from the raw data of both devices we computed classical balance parameters. Then, as both devices provide global indexes as indicators of balance abilities, we compared these global measures to assess whether those indexes carry similar information.

## Materials and methods

### Balance assessment

**Fig. 1:**
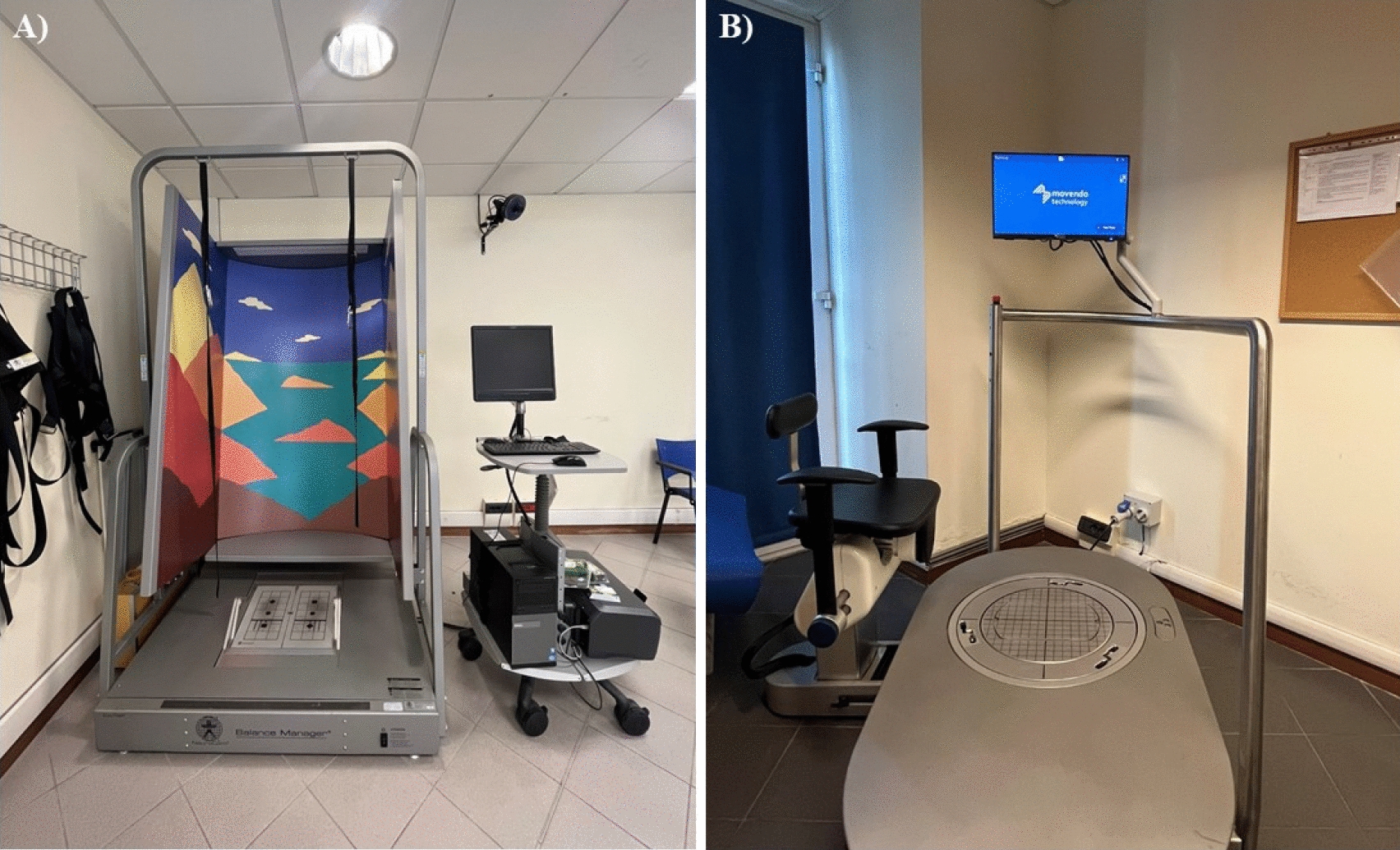
Devices used for the postural assessment. **A** EquiTest® from NeuroCom International, Inc., Clackamas, OR; **B** hunova® from Movendo Technology s.r.l., Genoa, IT

All the participants were assessed with both the EquiTest® and hunova® (Fig. 1A, B, respectively).

The EquiTest® (Fig. 1A) consists of a movable 46 × 46 cm dual force plate with two twin platforms connected by a pin joint oriented in the left–right direction, crossing the centre of the anterior–posterior axis. The two platforms can tilt simultaneously around the pin joint and glide in the anterior–posterior (AP) direction through a PC-controlled servomotor [25].

*Hunova®* (Fig. 1B) is a new medical robotic device aimed at giving a response to the clinical need for the functional sensory–motor evaluation and rehabilitation of the ankle, lower limbs and trunk that supports doctors, physiotherapists, and patients throughout assessments, treatments, and recoveries [23]. hunova® consists of two electromechanical and sensorized platforms with two degrees of freedom (forward/backward and left/right), one at the foot level and one at the seat level. This device enables the evaluation of balance while standing (both in mono- and bi-podalic configurations) and while sitting, both in static and dynamic conditions.

One assessor, a physiotherapist expert in MS, tested individuals’ static stability, i.e., the ability to maintain the position of the center of mass in unsupported stand when the base of support does not change, with the first two conditions of Sensory Organization Test (SOT) for static balance with EquiTest® and the Balance Test on static base (BT-sb) in hunova®. In both SOT and BT-sb, participants were required to stand upright, with their arms relaxed along the sides of the body, looking straight ahead and to refrain from moving their feet for the duration of the test. SOT and BT-sb established whether sway increases when visual cues are removed and determines how effectively the participant makes use of somatosensory input. Both tests were performed with both EO and EC and randomly presented to the participants.

More details about SOT from EquiTest® and BT-sb from hunova® are provided [please see Additional files 1 and 2, respectively].

### Outcome measures

Data analysis was based on the raw data recorded during the static trials executed with EquiTest® and hunova®. In particular, it included the trials of the first (i.e., EO) and second (i.e., EC) SOT conditions (COND1 and COND2, respectively) for EquiTest® and on the two trials of BT-sb performed with EO and EC for hunova®. EquiTest® returns within the outputs, the Antero-Posterior (AP) and Medio-Lateral (ML) coordinates of the COP sampled at 100 Hz. Differently, hunova returns the measures recorded by the single sensors at a sampling frequency of 30 Hz. In this case, we used data from the force torque sensors to compute the ML and AP components of the COP. Before the computation of the performance parameters, the COP data from the two devices were filtered with a 4th order Butterworth filter with a cutoff frequency of 12 Hz. All the algorithms for the data processing were performed by means of MATLAB (MathWorks, Natick, MA, USA).

#### Quantitative balance measures

EquiTest and hunova provide different outcomes. If hunova® makes COP-related balance measures easily available to the user, those were not directly available for the EquiTest®. For consistency in the analysis, the COP-related balance measures for both devices were computed from raw data. Specifically, from the COP records, the following parameters were computed:

- Sway area (SA): area of the 95% confidence ellipse of the statokinesigram of the COP (expressed as cm^2^). The 95% confidence ellipse can be defined as the surface that contains (with 95% probability) the individual points that make up the statokinesigram.
- Anterior–Posterior and Medio-Lateral oscillation ranges (APO and MLO ranges, respectively): extent of oscillations in the AP and ML direction, which are proportional to the instability of the subject. They are computed by looking at the maximum and minimum shift of the CoP coordinates in the two principal directions (expressed in cm).
- Total path length: total length of the trajectory of the COP (expressed in cm).
- ML and AP average speed (cm/s): total travelled distance in the two principal direction, divided by the trial duration.
- Root Mean Squared (RMS) distance: average distance of the COP from the mean COP position. It provides information about the variability or fluctuations of the COP over time (expressed in cm) [26].

All these indicators are proportional to the instability of the subjects: the greater the values, the lesser the subject’s ability to maintain balance.

For both devices, we also computed global balance metrics, that are normally provided as output global indexes as comprehensive measures of balance abilities. As those indexes are slightly different, in terms of both mathematical definition and meaning, we want to compare them to understand whether they carry the same information and/or highlight different aspects related to the ability to maintain a state of balance. Indeed, we compared the Equilibrium Score, the Somatosensory Score, and the Strategy Score, which are the classical output provided by EquiTest®, with the Somatosensory Index and the Static Balance Index of hunova®. In detail, from EquiTest®, we extracted the following metrics:

*1.* *Equilibrium Score* is an overall measure of balance quantifying postural stability during each of the SOT trials. It compares the AP sway during each trial to the theoretical sway stability limit of 12.5°. A subject swaying to the limits of stability will receive a very low score. It is expressed as percentage, where 100% represents perfect stability and 0% refers to poor stability.

It is computed as shown in the following equation:

1 $$\mathrm{Equilibrium\,score}= \frac{12.5^\circ -({\theta }_{ \mathrm{max}}- {\theta }_{ \mathrm{min}}) }{12.5^\circ }*100$$

where 12.5° represents the maximum normal postural sway of the Center of Gravity (COG) in the AP direction, and θ refers to the forward–backward (i.e., AP) lean of the angle of the COG computed according to the following equation:

2 $$\theta = {\text{arcsin}}\,\left( {\frac{{{\text{COG}}_{{{\text{AP}}}} }}{{0.5527*H}}} \right)\, - \,2.3^{^\circ }$$

where H is the subject’s height, the COG_AP_ refers to the AP displacement of COG and 2.3° is the so called “forward lean” of the angle of the COG. For more details on the computation, see [25].

*2.* *Somatosensory Score* reflects the subject’s ability to use input from the somatosensory system to maintain balance and is computed as the ratio of the Equilibrium Scores with EC and EO. The SOM determines how effectively the participant uses somatosensory input when visual cues are removed.

*3.* *Strategy Score* quantifies the relative amount of movement at the level of the ankles (ankle strategy) and the hips (hip strategy) used by the subject to maintain balance during each trial. Indeed, healthy subjects usually moves primarily about the ankle joints when the surface is stable and shift to hip movements as they become less stable. Thus, it is an indicator of hip/ankle strategy expressed as percentage, where a score of 100% indicates a pure ankle strategy, while 0% represents a strategy solely based on hip movements see [25]. The strategy score is computed the following equation:

3 $${\text{Strategy}}{\mkern 1mu} {\text{Score}} = \,\left( {1 - \frac{{{\text{SH}}_{{{\text{max}}}} - {\text{SH}}_{{{\text{min}}}} }}{{11.4}}} \right)\,*\,100$$

where SH_max_ and SH_min_ represent, respectively, the maximum and the minimum shear force and 11.4 kg is the difference between maximum and minimum shear force generated by individuals who only used the hip strategy to maintain balance on a narrow beam [25].

Differently, for hunova®, we calculated:

- *Somatosensory Index* provides information similar to the Somatosensory score by EquiTest® as it compares the EO and EC performance and is computed as the ratio of the SA with EC and the SA with EO.
- *Static Balance Index* is a measure depending on spatial information of the postural oscillation and on the variability of the postural oscillations, computed from both the COP, recorded through the platform, and the trunk, recorded through the IMU for both EO and EC. More precisely, this score depends on the eight following indicators: ML and AP oscillation range (mm), ML and AP RMS distance (mm), SA (mm^2^), ML and AP oscillation range of trunk movements (measured in degrees by the IMU gyroscope), and quantity of trunk movement (measured by the IMU accelerometer). More specifically, it is computed the following equation:
  4 $$\mathrm{Static\,Balance\,Index} = - \frac{1}{N} \sum_{i=1}^{N}\frac{{x}_{i}}{{Ref}_{i}}$$

where *N* is eight (number of balance measures we considered), *x*_*i*_ is the value of the *i*th balance measure, Ref_i_ is the normality value for the *i*th measure. The normality values are computed with healthy unimpaired subjects, and as it is well-known the continuous effect of age on postural control [27], we used four normality values depending on the age of the subjects. These values were computed for the following four age ranges: 18–39, 40–64, 65–75, and >75. Static Balance Index is definitively calculated by averaging the eight scores. It decreases with the worsening of balance.

#### Device agreeableness

The agreeableness level toward the use of EquiTest® and hunova® was evaluated through a 10-points numeric rating scale (1—very not agreeable; 10—very agreeable).

### Participants

PwMS were recruited among those followed as outpatients at the AISM Rehabilitation Service of Genoa. ﻿Inclusion criteria were: MS diagnosis according to revised McDonald criteria [28], age between 18 and 75 years, relapsing–remitting (RR) course, a disability level as measured by the EDSS [29] ≤ 6, stable phase of disease without relapses or worsening in the last 3 months, BBS score > 35 indicating ability to stand upright and walking with at least one support, and normal cognitive functioning as indicated by a Montreal Cognitive Assessment (MoCA) [30, 31] score ≥ 24. Exclusion criteria were: psychiatric disorders, significant visual impairment defined as a Visual System scoring more than 2 at the Functional Systems Score of EDSS and cardiovascular and/or respiratory disorders.

All study procedures and consent forms conformed to the ethical standards of the 2013 revised Declaration of Helsinki and were approved by the regional ethical committee (Comitato Etico Regionale (CER) Liguria, reference number: 36/2022-DB id 12144). The participants provided informed consent to participate in the study and to the publication of the results.

### Statistical analysis

Main descriptive statistics (mean, standard deviation) were used to analyze sample clinical characteristics. As we expected our data to be better described by a linear relation more than a monotonic one, Pearson’s r correlation was run for each COP-related measure and global balance index computed from EquiTest® and hunova®.Correlation coefficients ranging from 0.20 to 0.39 were considered as moderate, from 0.40 to 0.59 as relatively strong, from 0.60 to 0.79 as strong, and higher as very strong correlation [33, 34]. An independent *t* test was run to test agreeableness level from EquiTest® and hunova®. All p values were two-tailed and statistical significance was defined by alpha error < 0.05. Statistical analysis was performed with IBM SPSS Statistics software, 23.0.

## Results

### Demographic and clinical characteristics

Forty-six PwMS (32 females; mean age 52.17 ± 10.26 years, range 26–71 years) were recruited for the study. Clinical characteristics showed a mean EDSS of 3.9 ± 1.3 and a mean disease duration of 11.89 ± 8.72 years. BBS score was 49.31 ± 5.30.

### COP-related balance measures correlation

**Fig. 2:**
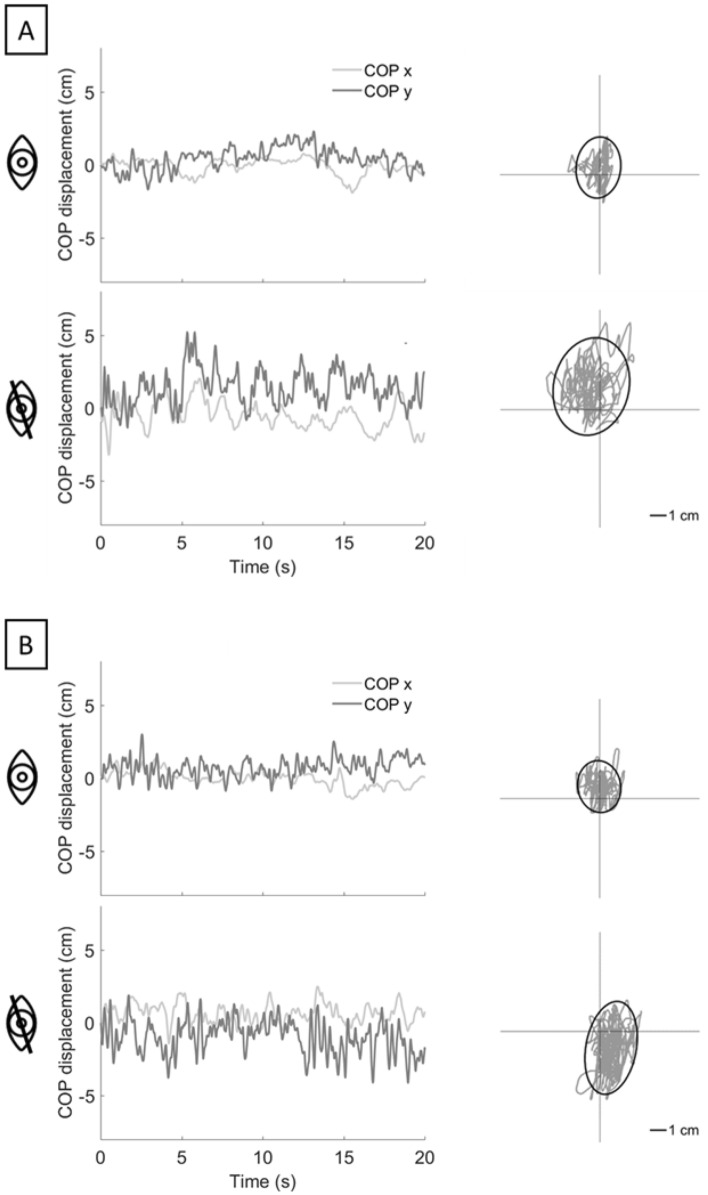
Stabilograms and statokinesiogram of one representative participant. CoP displacement calculated on hunova® (panel A) and EquiTest® (panel B) are shown. For stabilogram, light grey represents CoP x, while dark grey CoP y. In the statokinesigram, sway areas are represented

**Fig. 3:**
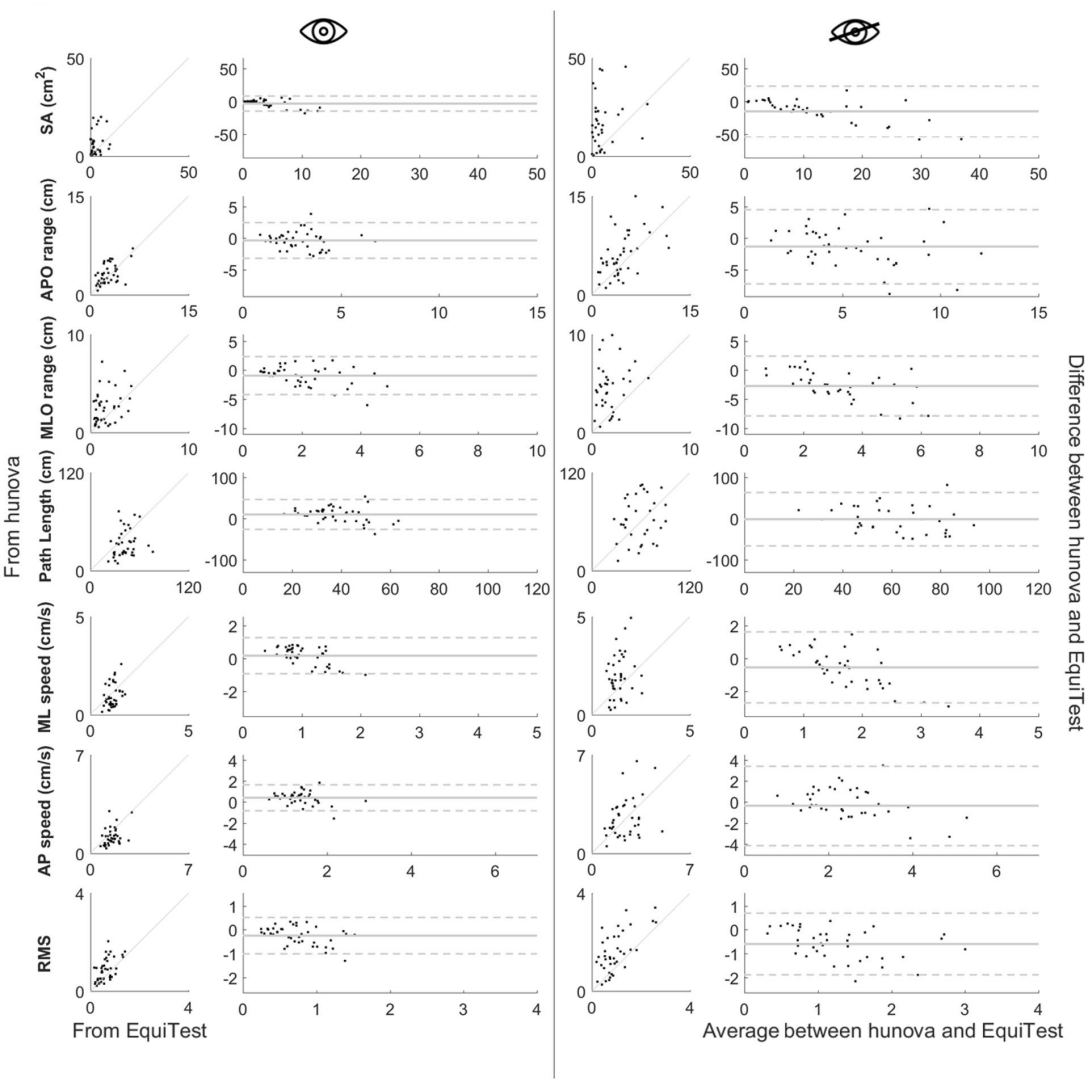
Graphical representation of all the parameters calculated in EquiTest® and hunova® for both EO (1st and 2nd columns) and EC (3rd and 4th columns)

Table 1 presents the mean value and the relative standard deviations for each parameter computed with both EquiTest® and hunova®, as well as the results of the correlation analysis, respectively, in the EO and EC condition. In addition, to deepen on this point, Fig. 2 shows the pooled stabilograms and statokinesiogram of one representative participant obtained from EquiTest® and hunova® for both the EO and EC condition. In addition, to be consistent with two previous work [26, 35], Fig. 3 shows the association of the parameters computed from EquiTest® and hunova® in the EO and EC conditions and the Bland–Altman plots which represent the relationship between the difference of the computed parameters with the two devices and its mean. Although there is not a complete match between the metrics computed from the two devices, particularly regarding SA and MLO range, it is noteworthy that the Bland–Altman plots do not reveal any apparent correlation between the mean and difference, which reinforces the statistically significant and overall good correlations reported in Table 1.

**Table 1:** Results of the Pearson’s r correlations between COP-related balance measures from EquiTest® and hunova® in the EO and EC conditions

	EquiTest®		hunova®		Pearson’s
	Mean	SD	Mean	SD	
*EO*					
SA	2.627	2.580	5.824	5.792	0.770
MLO range	1.550	1.100	2.503	1.628	0.595
APO range	2.746	1.368	3.147	1.517	0.485
Path Length	42.695	11.797	32.977	16.659	0.486
ML average speed	1.127	0.277	0.955	0.582	0.576
AP average speed	1.544	0.476	1.162	0.611	0.454
RMS distance	0.628	0.309	0.873	0.459	0.575
*EC*					
SA	6.080	6.742	21.060	19.654	0.645
MLO range	1.889	1.182	4.639	2.519	0.532
APO range	4.694	2.634	6.077	3.383	0.612
Path Length	60.035	20.701	61.787	26.683	0.750
ML average speed	1.396	0.432	1.949	1.095	0.603
AP average speed	2.435	0.937	2.814	1.911	0.782
RMS distance	0.977	0.599	1.576	0.835	0.614

*EO* eyes-open condition, *EC* eyes-closed condition, *SA* sway area (cm^2^), *MLO* medio-lateral oscillation range (cm), *AP* Anterior–Posterior oscillation range (cm); Path Length (cm), ML average speed: medio-lateral average speed (cm/s); AP average speed: anterior–posterior average speed (cm/s), *RMS* Root Mean Squared distance (cm). All ps < 0.001

More precisely, correlations were found significant for all the COP-related balance measures. Interestingly, in EO, SA presents a strong correlation (r = 0.770), while MLO and APO range, path length, ML and AP speed, RMS distance have a relatively strong correlation (0.454 ≤ *r* ≤ 0.576). In EC, except for MLO range that showed a relatively strong correlation (*r* = 0.532), other COP-related parameters present a strong correlation (0.603 ≤ *r* ≤ 0.782).

1st and 3rd columns represent the performance parameter computed from EquiTest® (*x*-axis) and hunova® (*y*-axis) for each single participant in the EO (1st) and EC (3rd) conditions. 2nd and 4th columns represent Bland–Altman Plots with both EO (2nd) and EC (4th) conditions. In detail, *y*-axis presents the difference between EquiTest® and hunova®, while *x*-axis presents the average between the two measures. The grey continuous line represents the mean difference between the devices, with the upper and lower lines representing the limits of agreement (2 standard deviation).

### Global balance indexes correlation

The results of the correlations on balance indexes computed from the scores of the EquiTest® and hunova® revealed a relatively strong correlation between the Somatosensory Score and the Somatosensory Index (*r* = − 0.488; *p* < 0.001), respectively. Please, note that the minus is expected, as the Somatosensory Score is computed from the Equilibrium Score, which is higher for good performance, while the Somatosensory Index is computed from the SA which is smaller for better performance. Although in EO Static Balance Index was highly correlated with Equilibrium Score (*r* = 0.416; *p* = 0.006), correlation between Static Balance Index and Strategy Score was moderate, with only a trend toward significance (*r* = 0.295; *p* = 0.054). Differently, in EC the Static Balance Index had a relatively strong correlation with both the Equilibrium Score (*r* = 0.482; *p* = 0.001) and the Strategy Score (*r* = 0.583; *p* < 0.001).

### Device agreeableness

Results from agreeableness numeric rating scale revealed that hunova® (7.09 ± 1.62) was highly appreciated than EquiTest® (6.33 ± 1.98) (*t* = − 2.075; *p* = 0.044), suggesting that PwMS preferred the novel robotic device compared to the traditional balance assessment tool.

## Discussion

Additional reliable and valid balance assessment devices can be extremely helpful in MS clinical settings. In recent years, the number of new technologies developed to provide a precise and complete assessment of balance has extremely increased [2, 36–38].

Thus, exploring the relationship between traditional technological performance-based measure and novel tool for balance assessment represents a key priority in scientific community [26, 39]. Cella and colleagues found that multidimensional balance parameters, as detected by the hunova® an innovative robotic platform, were significantly correlated with traditional tools that explore the reduction of physical performance in older persons [3, 40]. In this respect, for the first time, the postural assessment of PwMS during static conditions, with both open-eyes and closed-eyes, performed with hunova® and the gold standard EquiTest® was compared. We studied COP-related balance parameters and global balance indexes, extracted by hunova®, which resulted significantly correlated with those computed with a traditional and commonly used device in MS population as EquiTest®.

Despite the two devices are different in terms of sensors they use, the COP-related balance measures extracted from the raw data correlated. In addition, the correlation analysis on global balance indexes led to remarkable results. While the association between the Somatosensory Score and the Somatosensory Index was expected (both metrics compared the performance with EC with the one with EO), the other significant correlations found were quite unforeseen, as the other global indexes are relatively different, in terms of mathematical definition and meaning. Indeed, the EquiTest® provides two global indicators of balance, the Equilibrium Score, which is an overall measure of balance that compare the AP sway of a person with the theoretical limit (the closer to the limit you are, the worst your score), and Strategy Score, a measure highlighting whether a person is using a hip (low score value) or an ankle strategy (high value) [25]. Differently, the global score computed from hunova® compares the classical balance metrics with the normality. It includes both spatial information of the postural oscillation and its variability computed from both the COP, recorded through the platform, and the trunk, recorded through the IMU on the sternum [3]. Despite we expected a correlation between the Static Balance Index, that reflects information from feet platform and the IMU on the trunk by hunova®, with both the Equilibrium and the Strategy Score, which provide separately information about AP sway and ankle/hip strategy from EquiTest®, this hypothesis was not confirmed in the EO conditions. More precisely, with EO Static Balance Index was highly correlated with Equilibrium Score, but the correlation between Static Balance Index and Strategy Score was only moderate. Differently, in the EC condition we found a relatively strong correlation between the Static Balance Index and both the Equilibrium and the Strategy Score. One possible speculation is that the smaller correlation with the Strategy Score could be due to the low variability within subjects of the trial with EO [42], as it is the easiest and less challenging condition that do not require a large use of hip strategy. Taken all these results together, we can state that the information obtained with the EquiTest® in static condition are maintained during balance assessments with hunova®. In addition, the report provided by hunova® contains also all the classical balance metrics allowing all clinicians to examine the individuals’ performances directly and deeply [23]. To conclude on the information included in the reports of the two devices, the Equilibrium Score is overlooking an important aspect related to the risk of falling: the Equilibrium Score is only based on the AP sway, disregarding the sway in the ML direction, known to be highly related to falls risk [43], specifically in MS subjects [44, 45].

Despite significant correlations, intrinsic characteristics of devices and participants’ familiarity could have an impact on observed differences in COP-related measures from the two devices. Indeed, it is worth mentioning the numeric differences in terms of COP-related parameters. Figure 3 and Table 1 show that the bigger differences are in terms of Sway Area and MLO range. Differences in terms of numeric values have been found also in the study from [26], where they compared results from a Force Plate and the Wii Balance Board, which is a low cost technology that has been successfully proposed for balance assessment in both healthy [17, 18] and MS [11, 19] subjects. Having in mind that the mean EDSS in our study and [26] is slightly different, with subjects with higher EDSS in our sample (3.9 ± 1.3 and 3.4 ± 2.1, respectively), they found MLO range and Sway Area values that were in between the values we found for EquiTest® and hunoiva®. One possible explanation for this result is that EquiTest® tends to independently stabilize participants’ posture. During the assessment with the EquiTest®, subjects wear a safety harness, fixed to the safety bar, which prevents users from falling and lose or perceive to lose their balance. Furthermore, individuals may feel constrained by the surrounded EquiTest® limited space, while in hunova® participants perform exercises in a relatively open environment, since the only physical constraint is the monitor in front of them. This could also explain the higher satisfaction scores reported in favour of hunova® compared to EquiTest®, suggesting that EquiTest® may appear less appealing and then may prevent participants to join their routine assessment. Second, EquiTest® is a well-known balance tool available to PwMS followed at the AISM rehabilitation Service in Genoa since 2011, while hunova® is present on site from 2019, so we can speculate that the lower familiarization with the novel device could affect PwMS’ performances, leading to a major sway in COP-related parameters.

Designed as a robotic aid for physiotherapists that is intuitive and easy to use, the use of hunova® in different clinical settings such as neurology, orthopedics and geriatrics is enlarging and has been validated in several studies and clinical trials with promising results [23, 40, 46]. Furthermore, well-accepted by users, hunova® has been designed with the goal of maximizing the number of activities into a single device, providing attractive and numerous evaluation tasks and rehabilitative exercises for various components of balance. Given the high intra- and inter-variability of balance deficits in PwMS, hunova® offers huge potentialities for management of balance in MS population. One major advantage of utilizing hunova® rather than EquiTest® for assessing posture consists in the ability to objectively quantify the degree of postural motion in both AP and ML planes. This is particularly relevant since even small increases in sway in the ML direction, not investigated by EquiTest®, has been associated with falls in PwMS [2]. Consequently, it is of primary importance to assess balance skills in different relevant tasks (e.g., during standing and walking) and to assess patients’ perception of their own balance. Perception of balance may be an important factor in explaining the level of disability, because balance perception can have a direct consequence on patients’ behaviours.

In discussing our data, some important caveats need to be considered. Participants’ characteristics may limit the interpretation of our results. Study sample was constituted of individuals with a RR form, able to walk with at least one support (EDSS ≥ 6). Therefore, further attempts should be made to generalize findings to other individuals with MS as people with a progressive form or for those who require two walking aids as pair of canes or crutches (EDSS = 6.5). Second, for the purposes of the study, PwMS were tested only on static balance tasks with both open and closed eyes. hunova® allows testing balance under different conditions other than static one as: passive (e.g., movements of the platforms are pre-planned following given trajectories with different speed levels), active (e.g., the user can actively move the platforms, while it exerts a certain selectable resistance) and assistive (e.g., the device completes the exercise when subjects are unable to do it independently) modality (see Additional file 2). Given the huge potentialities provided by this innovative device, future studies should include outcomes from dynamic conditions, known to be more challenging than static ones, since they could provide a more meaningful and ecological information of balance in PwMS as they simulate situations commonly encountered in daily-life activities.

To conclude, since data from hunova® were found to provide important and distinctive information, further evidence are needed to explore whether this novel robotic tool could be sensitive for monitoring changes in balance over time as the disease progresses, and thus leading to a better evaluation of the effectiveness of tailored treatments for PwMS, thereby improving evidence-based clinical practice.

## Conclusions

In recent years, the use of robotic platforms has been growing rapidly thanks to novel computational approaches as well as sophisticated electronic components. In addition, for PwMS, technology-based solutions can provide more sensitive, specific and responsive monitoring for balance disorders. The present study confirms that hunova® can constitute an important innovative adjunct to traditional robotic balance assessment for PwMS, allowing for more sensitive monitoring of change in balance over time and a better evaluation of the effectiveness of treatment. This confirms that combining traditional and robotic assessments can more accurately identify balance impairments in MS.

## Supplementary Information

**Additional file 1. **Description of EquiTest® and Sensory Organization Test (SOT) for static balance assessment (NeuroCom International, Inc., Clackamas, OR)

**Additional file 2.  **Description of hunova® and Balance Test on static base (BT-sb) for static balance assessment (Movendo Technology s.r.l., Genoa, IT)

## Data Availability

The data sets used and analyzed during the current study are available from the corresponding author on reasonable request.

## Abbreviations

MS: Multiple sclerosis
PwMS: People with multiple sclerosis
RR: Relapsing–remitting
MoCA: Montreal Cognitive Assessment
M: Mean
SD: Standard deviation
EDSS: Expanded disability status scale
BERG: Berg balance scale
CoP: Center of pressure
COG: Center of gravity
SA: Sway area
APO: Anterior–posterior oscillation
MLO: Medio-lateral oscillation
AP: Anterior–posterior
ML: Mediolateral
SH: Shear force
RMS: Root mean square
IMU: Inertial movement unit
EO: Eyes-open
EC: Eyes-closed
SOT: Sensory organization test
