# Supplementary material for: Standard versus innovative robotic balance assessment for people with multiple sclerosis: a correlational study
Source: Eur J Med Res. 2023 Jul 26;28:254. doi: 10.1186/s40001-023-01223-2 (PMC10369743; doi:10.1186/s40001-023-01223-2)
Supplement: Supplementary file 1 — Additional file 1. Description of EquiTest® and Sensory Organization Test (SOT) for static balance assessment (NeuroCom International, Inc., Clackamas, OR) [file 40001_2023_1223_MOESM1_ESM.docx]

**Additional file 1**

The EquiTest® consists of a movable 46 x 46 cm dual force plate. The two twin platforms are connected by a pin joint oriented in the left–right direction, crossing the centre of the anterior–posterior axis. The two platforms can tilt simultaneously around the pin joint and glide in the anterior–posterior (AP) direction through a PC-controlled servomotor. Each EquiTest® plate has two sensors of vertical forces (A/D conversion: 100 Hz sampling rate, 12 bit) and a fifth transducer, sensitive to AP shearing forces, is bracketed to the centre plate directly beneath the pin joint.

EquiTest® allows the execution of standardized assessment protocols such as the Sensory Organization Test (SOT), Motor Control Test (MCT) and the Adaptation Test (ADT) that are standard protocols for the assessment of balance disorders, dizziness and mobility problems such as in MS^1^.

The SOT is a six-condition assessment able to isolate and quantify impairments in the patient’s use of somatosensory, visual, and vestibular inputs to balance, and impairments related to the patient’s use of specific sensory input when it is incorrect. Thus, it provides information about interactions among the three sensory systems contributing to postural control. The SOT also quantifies secondary maladaptive impairments related to the patient’s ability to select appropriate movement strategies and to accurately align their center of gravity (COG) relative to their base of support.

Each condition included 3 trials of 20 seconds. In Conditions 1 and 2 (COND-1 and COND-2), the participant stands quietly with eyes open and closed, respectively. This establishes whether sway increases when visual cues are removed and determines how effectively the participant makes use of somatosensory input. In Condition 3 (COND-3), the participant stands with their eyes open; the visual surround is sway-referenced and visual cues become inaccurate. In Condition 4 (COND-4), the support surface is sway-referenced; thus, somatosensory cues become inaccurate. Condition 5 (COND-5) is performed with eyes closed and a sway-referenced support surface. This determines how the participant makes use of vestibular cues when visual cues are removed and somatosensory cues are inaccurate. Finally, in Condition 6 (COND-6), the visual surround and support surface are both sway-referenced, which identifies if the participant relies on visual cues even when they are inaccurate^2^.

The SOT provides several analyses used to assess balance performances:

- Equilibrium score that quantifies postural stability during each of the three trials of six sensory conditions.
- Sensory analysis that reflects the sensory ratios computed from the average equilibrium scores in order to identify the subject’s ability to use input to maintain balance from somatosensory system (COND-2/COND-1), visual system (COND-4/COND-1) and vestibular system (COND-5/COND-1). Moreover the sensory analysis allows to establish the degree to which the subject relies on visual information to maintain balance, even when the information is incorrect ((COND-3 + COND-6)/(COND-2 + COND-5)).
- Strategy analysis that quantifies the relative amount of movement about the ankles (ankle strategy) and the hips (hip strategy) used by the subject to maintain balance during each trial.
- Center of Gravity alignment that reflects the subject’s center of gravity position relative to the support base center at the start of each SOT trial. People with normal performance maintain their center of gravity close to the center of the support base.

The MCT assesses the ability of the automatic motor system to quickly and effectively recover from unexpected support surface disturbances. The MCT isolates and quantifies impairments in the timing and strength of the automatic response in each leg; furthermore, it detects impairments in coordination of responses between the two legs and movement directions.

The ADT allows to assess the automatic motor system and to quantify impairments in the subject’s ability to adapt automatic responses to minimize sway when exposed to surface irregularities and unexpected changes in support surface inclination. The ADT also quantifies the subject’s capacity to systematically reduce their sway energy during repeated exposure to the same surface tilt disturbance.

The EquiTest® also assesses the Limits of Stability (LOS) that quantifies impairments in ability to intentionally displace the COG to the subject’s stability limits without losing balance, the Rhythmic Weight Shift (RWS) that quantifies the subject’s ability to perform rhythmic movements of their COG from left to right and forward to backward at three distinct paces, the Weight Bearing Squat (WBS) that quantifies the subject’s ability to perform squats with the knees flexed at 0°, 30°, 60°, and 90°, while maintaining equal weight on the two legs, and the Unilateral Stance (US) that quantifies the subject’s ability to maintain postural stability while standing on one leg at a time with the eyes open and closed.

1. NeuroCom International. NEUROCOM® INTERNATIONAL, INC. Balance Manager® Systems Technical Specifications. 2008;6744:1-8. https://www.neuroswiss.ch/view/data/5962/06-Dynamic_SMEQ_Package_with_LFP_INV.pdf.

2. Vanicek N, King SA, Gohil R, Chetter IC, Coughlin PA. Computerized Dynamic Posturography for Postural Control Assessment in Patients with Intermittent Claudication. 2013;(December):1-9. doi:10.3791/51077
