# Supplementary material for: Standard versus innovative robotic balance assessment for people with multiple sclerosis: a correlational study
Source: Eur J Med Res. 2023 Jul 26;28:254. doi: 10.1186/s40001-023-01223-2 (PMC10369743; doi:10.1186/s40001-023-01223-2)
Supplement: Supplementary file 2 — Additional file 2. Description of hunova® and Balance Test on static base (BT-sb) for static balance assessment (Movendo Technology s.r.l., Genoa, IT) [file 40001_2023_1223_MOESM2_ESM.docx]

**Additional file 2**

*hunova®* is a new medical robotic device aimed at giving a response to the clinical need for the functional sensory–motor evaluation and rehabilitation of the ankle, lower limbs and trunk that support doctors, physiotherapists, and patients throughout assessments, treatments, and recoveries ^1^. This device enables the evaluation of balance while standing (both in mono- and bi-podalic configurations) and while sitting, both in different testing conditions, such as static and dynamics; it has different difficulty levels for each task, and it integrates both visual and auditory feedback based on the user’s performance.

hunova® consists of two electromechanical and sensorized platforms with two degrees of freedom (forwards/backwards and left/right), one at the foot level and one at the seat level. The device operates in conjunction with a wireless 9-axis Inertial Movement Unit (IMU), including accelerometer, gyroscope and magnetometer to monitor trunk movements (i.e. located on the subject’s torso).

hunova® also comprises a tablet device which runs the clinician user interface. This graphical user interface (GUI) allows the operator to manage patients’ database, single patient’s training and assessment history and start exercise sessions directly from the tablet. The tablet is connected to the robotic system via a WiFi link ^2^.

As we mentioned before, hunova® allows testing balance under different conditions. More precisely, the device can simulate a static environment, or it can operate in a passive, an active and an assistive modality. In the passive modality, the movements of the platforms are pre-planned following given trajectories with different speed levels. In the active modality, the user can actively move the platforms while it exerts a certain selectable resistance. When the assistive modality is selected, the device completes the exercise when subjects are unable to do it independently.

Just to give a brief overview of the potentials of the device, in the following we list some of the trainings it enables:

- Balance tasks (both in mono- and bi-podalic configurations when standing, and while sitting)
  - on static platform
  - on unstable platform
  - on counter-resistance platform
  - on variable inclined plane
  - on a moving platform (following different trajectories)
  - on a platform giving impulsive perturbations
- Dual task: balancing on either static or moving platform while performing reaching tasks with the upper limbs (both in mono- and bi-podalic configurations when standing, and while sitting)
- Limits of stability tests (both in mono- and bi-podalic configurations when standing, and while sitting)
- Strengthening tasks, such as squats, isometric/isotonic and isokinetic tests
- Mobilization of the ankle
- Torso control
- Core stability tasks.

1. Movendo Technology. https://www.movendo.technology/our-products/hunova/?lang=en.

2. Saglia JA, Luca A De, Squeri V, et al. Design and development of a novel core, balance and lower limb rehabilitation robot: Hunova®. *IEEE Int Conf Rehabil Robot*. 2019;2019-June:417-422. doi:10.1109/ICORR.2019.8779531
